# Sphingosine-1-phosphate promotes the differentiation of human umbilical cord mesenchymal stem cells into cardiomyocytes under the designated culturing conditions

**DOI:** 10.1186/1423-0127-18-37

**Published:** 2011-06-07

**Authors:** Zhenqiang Zhao, Zhibin Chen, Xiubo Zhao, Fang Pan, Meihua Cai, Tan Wang, Henggui Zhang, Jian R Lu, Ming Lei

**Affiliations:** 1 Department of Neurology, Affiliated Hospital, Hainan Medical College, Haikou, 570102, PR of China; 2 Biological Physics Group, School of Physics and Astronomy, University of Manchester, M139PL, UK; 3 Cardiovascular and Genetic Medicine Research Groups, School of Biomedicine, University of Manchester, Manchester, M13 9NT, UK

**Keywords:** umbilical cord mesenchymal stem cells, sphingosine-1-phosphate, engineered cell sheets

## Abstract

**Background:**

It is of growing interest to develop novel approaches to initiate differentiation of mesenchymal stem cells (MSCs) into cardiomyocytes. The purpose of this investigation was to determine if Sphingosine-1-phosphate (S1P), a native circulating bioactive lipid metabolite, plays a role in differentiation of human umbilical cord mesenchymal stem cells (HUMSCs) into cardiomyocytes. We also developed an engineered cell sheet from these HUMSCs derived cardiomyocytes by using a temperature-responsive polymer, poly(N-isopropylacrylamide) (PIPAAm) cell sheet technology.

**Methods:**

Cardiomyogenic differentiation of HUMSCs was performed by culturing these cells with either designated cardiomyocytes conditioned medium (CMCM) alone, or with 1 μM S1P; or DMEM with 10% FBS + 1 μM S1P. Cardiomyogenic differentiation was determined by immunocytochemical analysis of expression of cardiomyocyte markers and patch clamping recording of the action potential.

**Results:**

A cardiomyocyte-like morphology and the expression of α-actinin and myosin heavy chain (MHC) proteins can be observed in both CMCM culturing or CMCM+S1P culturing groups after 5 days' culturing, however, only the cells in CMCM+S1P culture condition present cardiomyocyte-like action potential and voltage gated currents. A new approach was used to form PIPAAm based temperature-responsive culture surfaces and this successfully produced cell sheets from HUMSCs derived cardiomyocytes.

**Conclusions:**

This study for the first time demonstrates that S1P potentiates differentiation of HUMSCs towards functional cardiomyocytes under the designated culture conditions. Our engineered cell sheets may provide a potential for clinically applicable myocardial tissues should promote cardiac tissue engineering research.

## Background

Mesenchymal Stem cells (MSCs) are pluripotent cells that are able to differentiate into various specific cell types. Because of their plasticity, MSCs have been suggested as potential therapies for numerous diseases and conditions. *In vitro *differentiation of MSCs into cardiomyocytes offers a new cellular therapy for heart diseases. Therefore, it is of growing interest to develop novel approaches to initiate differentiation of various types of MSCs into cardiomyocytes. Human umbilical cord (UC) has been a tissue of increasing interest for such purpose due to the MSCs potency of stromal cells isolated from the human UC mesenchymal tissue, namely, Wharton's jelly[1]. A number of recent studies have shown that HUMSCs are able to differentiate towards multiple lineages including neuronal and myocardiogenic cells *in vitro*, thus providing a great potential for cell based therapies and tissue engineering for heart diseases[1-3].

However, differentiation of MSCs into specific cell types is a complex biologic process involving a sequence of events and cellular signalling pathways that are still poorly understood. To understand the cellular signalling for differentiation of MSCs has been one of the research focuses in MSCs research. Sphingosine-1-phosphate (S1P), a key member of Sphingolipids, is a circulating bioactive lipid metabolite that has been known for many years to induce cellular responses, including proliferation, migration, contraction, and intracellular calcium mobilization. Recent Evidence indicated that S1P can function as an intracellular second messenger implicating them in physiological processes such as vasculogenesis. Interestingly, recent evidence has also demonstrated that S1P has potent effects on the embryonic and neural stem cell biology such as differentiation, proliferation and maintenance[4-6]. Based on these results, we speculate that S1P could have a potential to affect biology of MSCs derived cardiomyocytes. Thus, the aims of the present study are two folds; firstly, to determine whether S1P can promote differentiation of HUMSCs towards functional matured cardiomyocytes under the designated culture conditions; secondly, to develop an engineered cell sheet from HUMSCs derived cardiomyocyte with potential clinical application by using temperature-responsive polymer, poly(N-isopropylacrylamide) (PIPAAm) cell sheet technology.

## Methods

### Cell culture

Human cardiac myocytes (HCM, Cat. No. 6200) were purchased from ScienCell Research Laboratories (San Diego, CA, USA). The cells were initially expanded in 75 cm^2 ^flasks (NUCN, Cat. No.156499) pre-coated with poly-L-lysine (2 μg/cm^2^) by using culturing medium consisting of 500 ml of basal medium, 25 ml of fetal bovine serum (ScienCell Research Laboratories, Cat. No. 0025), 5 ml of cardiac myocyte growth supplement (Cat. No.6252) and 5 ml of penicillin/streptomycin solution (Cat. No.0503). All cells were maintained at 37°C in humidified air with 5% CO_2_. Cellular growth was monitored every day by inspection using phase-contrast microscopy. The medium was changed every other day. The cells were sub-cultured when they were over 90% confluence.

HUMSCs were also purchased from ScienCell Research Laboratories (San Diego, CA, USA). The cells were also initially expanded in 75 cm^2 ^flasks (NUCN, Cat. No.156499) precoated with poly-L-lysine (2 μg/cm^2^) with culturing medium consisting of 500 ml of basal medium, 25 ml of fetal bovine serum (ScienCell Research Laboratories, Cat. No. 0025), 5 ml of mesenchymal stem cell growth supplement (Cat. No.7552) and 5 ml of penicillin/streptomycin solution (Cat. No.0503). All cells were maintained at 37°C in humidified air with 5%CO_2_. Cellular growth was monitored every day by phase-contrast microscopy.

### Preparation of cardiac myocyte condition medium

The cardiac myocytes conditioned medium (CMCM) was prepared in T-75 flasks by culturing cardiomyocytes in DMEM (D 6429 Sigma-Aldrich, St. Louis, MO) and 10% FBS. When the cardiac myocytes were over 50% confluence, the medium was then collected and centrifuged at approximately 800 g for 10 minutes at room temperature, and the supernatant was filtered for use as conditioned medium.

### Cardiac Differentiation

After 5-8 passages, HUMSCs were plated on poly-L-lysine coated coverslips in 24-well plates at the density of 1 × 10^3 ^cells/cm^2 ^in DMEM +10%FBS and grown to adherence. They were then cultured in different conditional mediums including cardiac myocytes condition medium (CMCM) plus 1 μM S1P or cardiac myocytes condition medium or DMEM +10% FBS plus 1 μM S1P. The medium was changed every 3 days. Cardiac differentiation of HUMSCs was assessed at different time points by morphology and immunostaining with cardiac myocyte specific markers.

### Immunocytochemistry

The medium was first removed and the cells were washed twice with PBS, fixed for 30 min with 4% paraformaldehyde. Cells were permeabilized for 20 min with 0.1% Triton X-100 and then blocked for 30 min in 5% normal goat serum. Cells were then incubated with the primary antibody (Ab) (either mouse anti-α-actinin (sarcomeric) at a dilution of 1:200, or mouse anti-myosin cardiac heavy chain α/β at a dilution of 1:4 (Millipore, Billerica, MA, USA) in PBS-1% BSA overnight at 4°C. Excess primary antibody was removed by a triple wash in PBS, and the cells were then incubated with secondary Ab (Rhodamine-conjugated anti-mouse IgG (Millipore, Billerica, MA, USA), at dilutions of 1:100 in PBS at room temperature for 1 h. After washing three times with PBS-1% FBS, the coverslips were mounted onto glass slides in Vectashield (Vector Laboratories, Burlingame, CA, USA). Examination of the slides was performed using a confocal microscope equipped with a digital camera. Negative control (omit primary antibody) was included in all immunofluorescent staining. Immunolabelled cells were viewed using Zeiss LSM 510 laser scanning confocal microscope (Zeiss Ltd, Jena, Germany) equipped with argon and helium-neon lasers, which allowed excitation at 550 nm wavelengths for the detection of Rhodamine at 570 nm, respectively. All images presented are single optical sections. Images were saved and later processed using Zeiss LSM Image Bowser (Zeiss Ltd).

### Electrophysiological measurement

Electrophysiological measurements were performed on human UC-MSC-derived caridomyocytes in S1P+CMCM and CMCM groups. According to the results of immunostaining, the cardiomyocyte-like cells were chosen at co-culture time point of 10 days. For electrophysiological recordings, the cells were grown on glass coverslips at the density that enabled single cells to be identified. Whole-cell currents were recorded using the patchclamp technique, a 200B amplifier (Axon Instruments, Foster City, CA, USA), and with patch pipettes fabricated from borosilicate glass capillaries (1.5 mm outer diameter; Fisher Scientific, Pittsburgh, PA, USA). The pipettes were pulled with a PP-830 gravity puller (Narishige, Tokyo, Japan), and filled with a pipette solution of the following composition (in mmol/L): CsCl 130, NaCl 10, HEPES 10, EGTA 10, pH 7.2 (CsOH). Pipette resistance ranged from 2.0 to 3.0 MΩ when the pipettes were filled with the internal solution. The perfusion solution contained (in mmol/L): NaCl 140, KCl 4, CaCl_2 _1.8, MgCl_2 _1.0, HEPES 10, and glucose 10, pH 7.4 (NaOH). Series resistance errors were reduced by approximately 70-80% with electronic compensation. Signals were acquired at 50 kHz (Digidata 1440A; Axon Instruments) and analyzed with a PC running PCLAMP 10 software (Axon Instruments). All recordings were made at room temperature (20-22°C).

### Synthesis of thermo-responsive copolymer, film coating and characterization

#### Chemicals

N-isopropylacrylamide (NIPAAm, 98% pure) was purchased from Sigma-Aldrich and was freshly recrystallized in hexane, followed by freeze-drying before use. Hydroxypropyl methacrylate (HPM) and 3-trimethoxysilylpropyl methacrylate (TMSPM, the cross-linking agent) were purchased from Aldrich and used as supplied. The initiator 2, 2-azobisisobutyronitrile (AIBN) was purchased from BDH (UK) and was fully recrystallised in ethanol followed by freeze-drying before use. The solvents including ethanol, acetone and n-hexane were all above 99% pure (Aldrich) and used as supplied. Water used was processed using Elgastat ultrapure (UHQ) system. The silicon wafers were purchased from Compart Technology Ltd (UK) and were cut into 1 × 1 cm^2 ^cuts before use. They were cleaned by 5% (v/v) Decon 90 solution (Decon Laboratories), followed by rinsing with UHQ water and dried. The glass coverslips with diameter of 13 mm were purchased from VWR (Belgium). All plastic vessels (except those for single use in cell culture) were cleaned by soaking them in 5% Decon solution. All glassware was immersed into piranha solution (H_2_O_2_: H_2_SO_4 _= 1:3 by volume) for 30 min, followed by abundantly rinsing with tap water and UHQ water.

#### Synthesis of the Copolymer

Poly(N-isopropylacrylamide) copolymer (PNIPAAm) was synthesized by free radical polymerization following the procedures as reported with modifications[7-9]. Monomers of NIPAAm (2 g), HPM (0.13 g) and TMSPM (0.22 g) were kept at the molar ratios of 1:0.05:0.05. These samples together with 10 ml of absolute alcohol were added into a three necked flask with a condenser, and subsequently purged with nitrogen for about 10 min. 1 mol% of the total (NIPAAm + HPM + TMSPM) of AIBN was added into the mixture solution (0.0319 g). The mixture was then kept under heating and stirring at 60°C overnight under nitrogen protection. The solvent ethanol was then evaporated and a small amount of acetone was then added into the remaining sample to dissolve it. The liquid was then added drop wise into n-hexane for precipitation. The precipitation process was repeated three times using acetone as solvent and n-hexane as non-solvent. The product was then dried at -60°C in the vacuum freeze dryer and stored in a refrigerator for use. Both FTIR and NMR studies confirmed the structure and composition of the copolymer.

#### Film formation and characterization

The PNIPAAm copolymer was dissolved into absolute ethanol at 1 or 2 mg/ml. The solution was then used to form PNIPAAm copolymer films by spin coating using a single wafer spin processor (Laurell Technologies, North Wales) at 3000 rpm and the spin coating time of 20 s. The coated films were dried in air for at least 30 min and then annealed for 3 h at 125°C under vacuum to facilitate 3-trimethoxysilyl cross-linking and reacting with hydroxyl groups, and to remove the residual solvent. Any un-reacted monomers and unconnected copolymers were extracted by soaking and washing the wafers or coverslips in ethanol and water thoroughly. The thickness of the coated copolymer films was determined from films coated onto optically flat silica wafer, thus facilitating spectroscopic ellipsometry (Jobin-Yvon UVISEL, France). Upon the use of refractive index of 1.47 for the copolymer, the dry films were found to be between 3-5 nm. For cell culturing, the copolymer films were coated onto glass cover-slips suitable for placing into the wells of 24-well cell culture plate and undertaking microscopic observation.

### Culturing and thermo-responsive detachment of cell sheets

The glass coverslips coated with PNIPAAm copolymer films were sterilized for 1 h by UV and then transferred into 24 well tissue culture plates for subsequent use. Some of the glass coverslips were half coated so that the bare glass surfaces worked as control. Before starting cell culture, the coverslips were rinsed repeatedly with PBS and the cells were planted on the coverslips immersed in medium as described above, at the density of 1.0 × 10^4 ^cells/well and cultured for 6-7 days at 37°C in humid air with 5% CO_2_. Cell growth status and morphology was observed by inverted phase contrast microscope (TE2000-U, Nikon). The number of adhesive cells was counting by hematocytometer. After aspiration of outspent medium, the cold fresh culture medium (less than 20°C) was introduced accompanied by gently pipetting. The assessments focused on cell growth under culture condition at 37°C and the extent of detachment at 20°C. It was found that films coated at 1 and 2 mg/ml provided healthy growth and swift detachment of cell sheets when the 24-well plates were taken out of the 37°C incubator and left for cooling at 20°C. Gentle scratching around the edge of the glass coverslip was made using a micropipette tip to help separate the cell sheet from the wall of the culturing well. Gentle squeezing of culture fluid against the confined cell sheet using the micropipette tip was also helpful to aid its detachment from the thermo-responsive surface. Standard MTT assays were used to assess HCM cell viability using glass coverslips, tissue culture plastic wells and poly-L-lysine coated surfaces as controls.

### Statistical analysis

Results are presented as mean ± standard error of the mean (SEM). Statistical analyses were performed using the one-way ANOVA test with significance being assumed for p < 0.05.

## Results

### Morphological changes of HUMSCs under designed cardiomyocyte culturing condition induction

**Figure 1:**
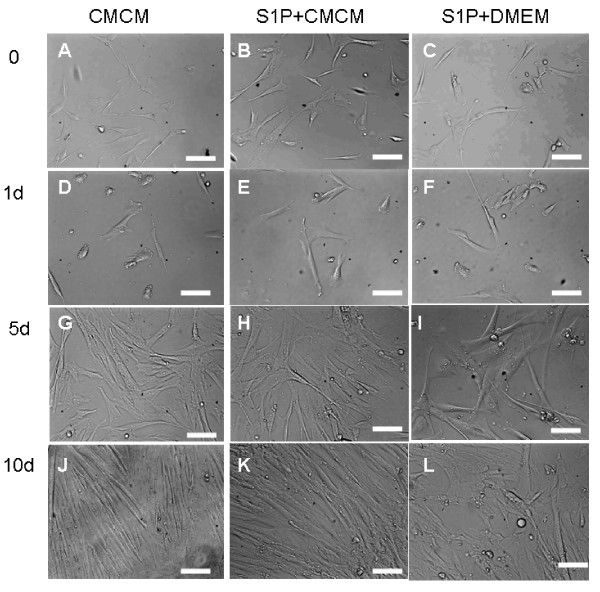
**UC-MSC cells showed a fibroblast-like morphology before conditioned culturing (AC); the induced cells change their morphology with time**. In cells treated with CMCM or CMCM+S1P, HUMSCs displayed a cardiomyocyte-like morphology such as myotube-like shape between 5-7 days (D, E, G, H); At around 10 days, the cells became elongated and lined up in CMCM and CMCM+S1P groups (J, K), and the alignment of the cells appeared in an ordered perpendicular terrace-pattern, like intercalated disc in CMCM+S1P groups. (K). But the cells had no similar change in S1P+DMEM groups (F, I), and the alignment looked random. (L)

We first attempted cardiomyogenic differentiation of HUMSCs by culturing these cells with different conditioned mediums. HUMSCs, after 5-8 passages, were seeded onto poly-Llysine coated coverslips in 24-well plates at the density of 1 × 10^3 ^cells/cm2 in DMEM+10%FBS and grown to adherence. They were then sub-cultured in either CMCM alone or CMCM plus 1 μM S1P; or DMEM+10%FBS+1 μM S1P. Medium was changed every three days. The morphological changes of HUMSCs during cardiomyocyte induction were monitored. Figure 1 shows phase contrast photographs from HUMSCs cells at the start and after being subject to the conditioned culturing for 1, 5 and 10 days with different conditioned mediums. HUMSCs showed a fibroblast-like morphology before conditioned culturing (Figure 1A-C), and this phenotype was retained through repeated subcultures under non-stimulating conditions. After induction with conditioned culturing (Figure 1D-K), the cells began to change their morphology with time. In cells treated with CMCM or CMCM+S1P, HUMSCs displayed a cardiomyocyte-like morphology such as myotube-like shape between 5-7 days after induced culturing. At around 10 days, the cells became elongated and lined up in CMCM and CMCM+S1P groups, the differentiated myotubes showed a number of branches, but the cell group under DMEM aligned randomly.

### Immunocytochemical analysis and patch clamping confirmed cardiomyogenic differentiation and maturation

**Figure 2:**
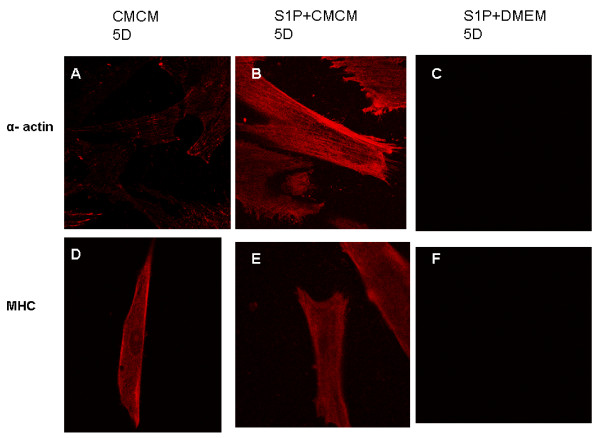
**Immunostaining of anti-α-actinin and anti-α MHC in cells at different time points of culturing**. A strong expression of both α-actinin and MHC proteins (A, B, D, E, G, H, J, K) was observed in CMCM and CMCM+S1P groups, but not in cells from the DMEM+S1P group(C, F, I, L).

**Figure 3:**
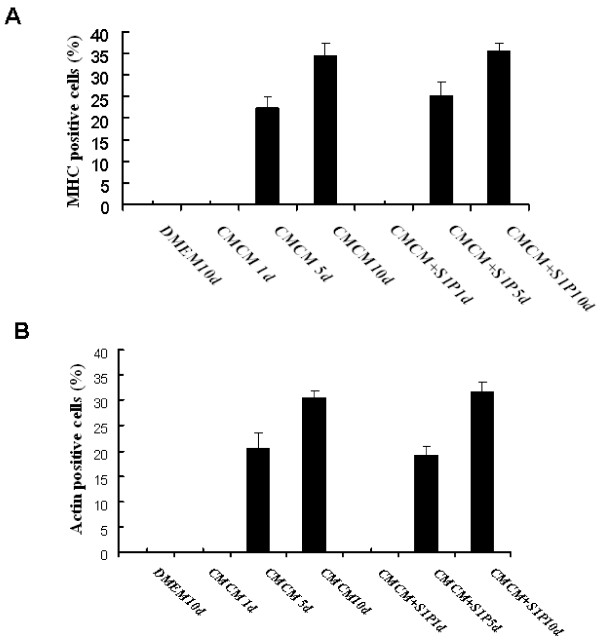
**Histograms showing the percentage of human umbilical mesenchymal cells expressing α-actin (A) and sarcomeric α/β myosin cardiac heavy chain (B) after CMCM or CMCM+S1P treatment**. The results are expressed as mean ± SE of ten randomly selected microscopic fields each from two different experiments. At least 200 cells were counted in each experiment. A statistical difference at *P < 0.05 compared with DMEM-only group and 1 day; *P < 0.05 compared with 5 days. B statistical difference at *P < 0.05 compared with DMEM-only group and 1 day; *P < 0.05 compared with 5 days.

Cardiomyogenic differentiation and functional maturation were then determined by immunocytochemical analysis of the expression of cardiomyocyte markers and patch clamping recording of the action potential and voltage gated membrane currents. Immunostaining with specific antibodies revealed that cardiomyocyte markers including myosin heavy chain (MHC) and sarcomeric α-actinin were strongly expressed in differentiated myocardiomyocytes in CMCM and CMCM+S1P groups. Figure 2A-C, G-I represents the fluorescent immunostaining of α-actinin of cells from three groups, while, J-L shows the fluorescent immunostaining of MHC of cells from these groups after 5 and 10 days' culturing. Cells from CMCM and CMCM+S1P groups show strong expression of both α-actinin and MHC proteins, but not those cells from DMEM+S1P group. Figure 3 shows the time dependent expression and the percentage of cells expressing α-actinin and sarcomeric α/β myosin cardiac heavy chain after CMCM or CMCM+S1P treatment. A significant increase in expression of both markers after 5 days culturing was observed in both groups.

**Figure 4:**
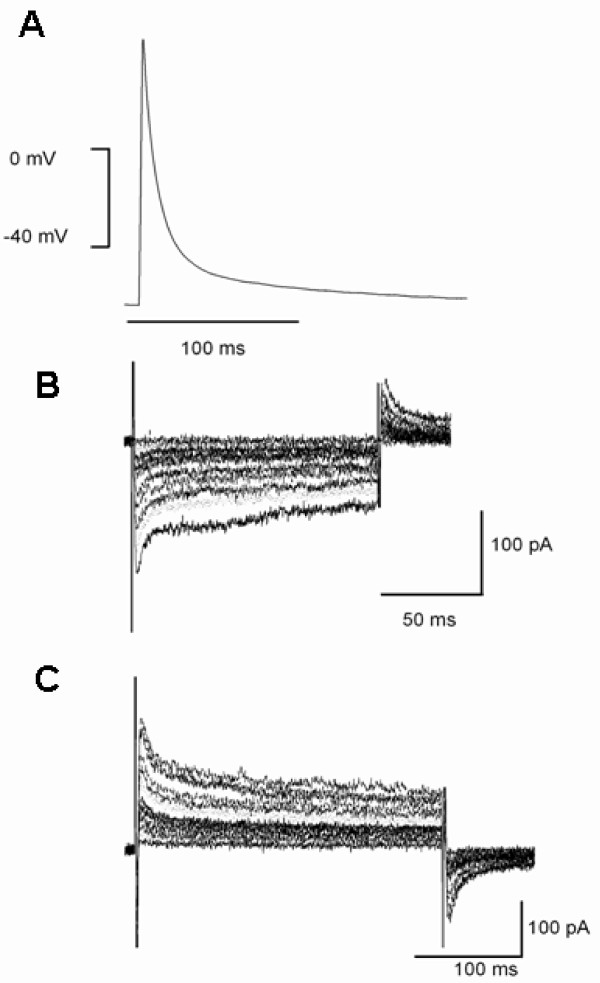
**Representative recordings of action potential (A) and whole cell voltage gated inward (B) and outward currents (C) by whole cell patch clamping in myocytes of CMCM+S1P group**. The currents were recorded during 200 ms step depolarization pulses from a holding potential of -50 mV to a range of potential between -40 mV and +50 mV.

Figure 4 shows representative examples of action potential and voltage dependent currents recorded from myocytes of CMCM+S1P group. A rapid upstroke, with lack of plateau phase action potential (Figure 4A), was recorded from cells in CMCM+S1P group. Such features were not observed from the cells in CMCM group. Furthermore, a voltage dependent inward current (Figure 4B) and a voltage dependent outward current (transient outward like current) (Figure 4C) can be recorded from the cells that displayed such action potentials.

### Formation and visualization of cell sheets

To explore the therapeutic potential, we then developed engineered cell sheets from a polymer coated cell culturing substrate. The thermo-responsive films were coated onto glass coverslips, which were then placed into the wells of 24-well plates after thermal annealing, cleaning and sterilization. Cell culturing was undertaken using surfaces coated with 1 and 2 mg/ml solution and parallel studies using bare tissue culture plastic surfaces (TPCS), glass coverslips (G), coverslips adsorbed with polylysine (G+L), G+L surface adsorbed with CM medium protein (G+L+CM).

Cell adhesion was assessed by washing the loosely attached cells through rinsing with buffer after 24 hr culturing. The percentages of cells attached to thermo-responsive surfaces with and without poly-L-lysine adsorption were between 80 and 83%; those on the bare TPCS was just about 80% and those on the bare glass substrate were between 78 and 80%. Cell morphological observations indicated that after 2 days of culturing, there were little visual differences between cells grown on different surfaces. However, on G+L+CM surface, cell numbers appeared to be greater. GFP transfection showed no visible effects arising from surface coating on the shape or morphology of the cells. Hoechst 33258, a specific DNA dye that binds the A-T bonds, could reveal nuclear fragments indicating apoptosis. Under a fluorescence microscope, live cells show smooth, weak but visible light; dead cells do not show colour, but when cells enter apoptosis,, the cell nuclei and cytoplasm show stains, usually in the form of small lumps and an abnormal nuclear shape. If there are 3 or more fragments or lumps, the cell is regarded as undergoing apoptosis. No indication of cell apoptosis was noticed from the PNIPAAm coated surfaces. These analyses thus concluded that the thermo-responsive coated film surfaces did not cause any adverse effects on cell viability and phenotype.

**Figure 5:**
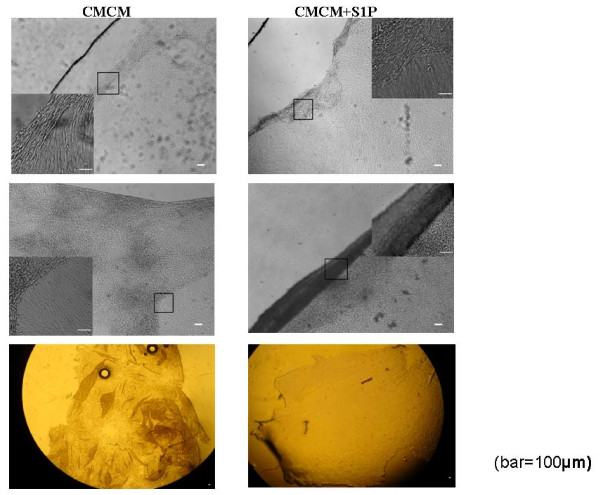
**Human cardiac myocyte cell film (left) or differentiated HUMSCs (right) in CMCM+S1P group detachment under cooling at the ambient temperature of 20°C**. Top panel shows the local detachment, middle panel shows a large cell sheet peered off and the bottom panel shows a large pile of cell sheets.

Cell sheets or films can be separated from the culturing surface by cooling down to the ambient temperature, placing the plates in a 4°C fridge for 2-3 minutes or adding cold cell culture medium to speed up. Films came off from 10 to 30 minutes upon cooling. Free cell films could be cut and transported to different surfaces. A few examples of detached or partially detached cell films are shown in Figure 5.

## Discussion

A number of studies have shown that HUMSCs are able to differentiate towards multiple lineages under *in vitro *conditions including adipocytes, osteoblasts, chondrocytes, skeletal myocytes, cardiomyocytes, neurons, and endothelial cells[1-3]. Given these characteristics, particularly the plasticity and developmental flexibility, UC stromal cells are now considered an alternative source of stem cells and deserve to be examined in long-term clinical trials, to enable the potential use of HUMSCs for cell based therapies and tissue engineering for heart diseases. Differentiating HUMSCs into cardiomyocytes was less examined and the functional characteristics of HUMSCs differentiated cardiomyocytes have not been reported so far.

In the present study, we demonstrated that cardiomyocytes can be induced from HUMSCs by designed conditional culturing alone or with conditional culturing combined with S1P. As demonstrated in Figure 1, after induction with conditioned culturing, the cells began to change their morphology with time. In cells treated with CMCM or CMCM+S1P, HUMSCs displayed a cardiomyocyte-like morphology such as myotube-like shape between 5-7 days after induction of culturing. At around 10 days, the cells became elongated and lined up in CMCM and CMCM+S1P groups. In the S1P+CMCM group, the alignment of cells appeared in an ordered perpendicular pattern, like intercalated disc. Our results indicate that conditioned culturing is the basis for cardiomyocyte induction of HUMSCs. However, S1P potentiates the differentiation, but alone cannot lead to cardiomyocyte induction of HUMSCs. Such findings provide a potential role for S1P in causing cardiomyocyte induction of HUMSCs under *in vivo *conditions and should be an exciting direction to explore in the future.

As demonstrated in Figure 2, Immunostaining with specific antibodies revealed that cardiomyocyte markers including myosin heavy chain (MHC) and sarcomeric α-actinin were strongly expressed in differentiated myocytes in CMCM and CMCM+S1P groups. While both CMCM and CMCM+S1P groups develop cardiomyocyte-like cells, identified morphologically and molecularly, only cells from CMCM+S1P group show electrophysiological characteristics of cardiomyocytes with an atrial type of AP and major voltage gated inward and outward currents. This suggests that S1P triggers differentiation of HUMSCs into cardiomyocytes and maturation of HUMSCs derived cardiomyocytes.

Admittedly, the detailed mechanism(s) of above effects of S1P on differentiation of and maturation of HUMSCs derived cardiomyocytes requires further investigation. S1P is a bioactive Lysophospholipid and signals both extracellularly, through EDG (Endothelial Differentiation Gene) receptors (called S1P receptors) coupled to three heterotrimeric G proteins, G_i_, G_12/13_, and G_q_, and intracellularly by undefined mechanisms. S1P has been known to implicate in a diverse range of biological processes, including cell growth, differentiation, migration and apoptosis in many different cell types. A number of recent studies provided several lines of evidence to indicate that S1P signals involved in biology of MSCs. Avery et al demonstrated that S1P plays an important role in survival and proliferation of hESCs, and found that the key signaling pathways and downstream targets of S1P were investigated in a representative cell line hESCs-Shef 4[4]. A significant rise in ERK1/2 activation with S1P treatment was witnessed in hESCs maintained on murine embryonic fibroblasts (MEFs) exhibiting significantly higher levels of active ERK1/2 than those grown on Matrigel. S1P regulated apoptosis through several BCL-2 family members, including BAX and BID, with increased expression of cell cycle progression genes associated with proliferation of hESC cultures. He et al [10] recently further investigated the role of S1P in the growth and multipotency maintenance of human bone marrow and adipose tissue-derived MSCs. They showed that S1P induces growth, and in combination with reduced serum, or with the growth factors FGF and platelet-derived growth factor-AB, S1P has an enhancing effect on growth. The results demonstrated that S1P is able to induce proliferation while maintaining the multipotency of different human stem cells. Our investigation indicates that S1P promotes differentiation of HUMSCs towards cardiomyocytes and functionally maturation of hUC-MSCs derived cardiomyocytes, such role could be through S1P receptors coupled to heterotrimeric G proteins and intracellularly by undefined mechanisms.

Myocardial tissue engineering has now emerged as a promising treatment for heart diseases such as severe heart failure. As a new transplantation therapy, "cell sheet engineering" has been developed over the past decade. Several types of myocardial tissues have been successfully engineered by seeding cells into poly (glycolic acid), gelatin, alginate or collagen scaffolds[11]. For examples, Shimizu and coworkers showed that poly-surgerical approach based cell sheet integration appears feasible for fabricating viable, thick heart tissues with appropriate vascular network formation and without mass transport limitations[11]. Wang and coworkers also have injected MSCs sheet fragments with ECM into myocardial infarction area to improve the efficacy of therapeutic cells[12]. Several previous reports have utilized the live growth of a temperature-responsive polymer, poly(N-isopropylacrylamide) (PIPAAm) from its monomer under electron beam irradiation (e.g., 0.25 MGy electron beam dose) to form temperature-responsive culture surface. In the present study, we developed a new approach to form PIPAAm based temperature-responsive culture surfaces. Instead of undertaking live surface polymerization, our approach involved the easy first step of coating an already made N-isopropyl acrylamide containing copolymer and the second step of annealing to induce cross-linking within the film and with the glass substrate for film stability. Subsequent cell culturing experiments have successfully produced both neonatal cardiac myocyte and cardiomyocytes sheets from differentiated human umbilical cord mesenchymal stem cells. We assessed viability of the cells of sheets at room temperature. No indication of cell apoptosis was noticed from the PNIPAAm coated surfaces. These analyses thus concluded that the thermo-responsive coated film surfaces did not cause any adverse effects on cell viability and phenotype. Further experiment on the survival and characteristic structures of the cardiomyocyte sheets in vivo is required. The new engineered cell sheets offers potential for clinically applicable myocardial tissues and should promote cardiac tissue engineering research exploiting the tissue fabrication utilizing ready-made cell sheets.

## Conclusions

In the present study, We demonstrated that S1P play a key role for differentiation of HUMSCs towards functional cardiomyocytes under t cardiac myocytes conditioned medium conditions. Utilizing the technology of HUMSCs cell sheets, we might find a way for treating myocardial diseases. However, although functional cardiomyocytes have been obtained from HUMSCs in this study, significant challenges remain in optimizing these cell preparations for experimental and potential clinical applications. The heterogeneity of cell types produced in differentiation protocols can be great even if one succeeds in isolating cardiomyocytes. For example, using a mixed population of cardiomyocytes in attempts at left ventricular repair raises concerns for proarrhythmia effects. Likewise, a preparation including undifferentiated cells could lead to tumorigenesis. Thus, approaches to produce homogenous or well characterized cell preparations remain a great need.

## Competing interests

The authors declare that they have no competing interests.

## Authors' contributions

ZZ carried out the cell culture, cardiac differentiation, immunocytochemistry. ZC conceived of the study, and participated in its design and coordination. XZ carried out Synthesis of the rmo-responsive copolymer, film coating and characterization. FP carried out the Culturing and thermo-responsive detachment of cell sheets. MC and TW carried out the collection and assembly of data, data analysis. HZ participated in the design of the study. JRL participated in the design of the study and coordination. ML participated in the design of the study and coordination and performed the Electrophysiological measurement. All authors read and approved the final manuscript.
